# Real‐life data from standardized preanalytical coding (SPREC) in tissue biobanking and its dual use for sample characterization and process optimization

**DOI:** 10.1002/cjp2.305

**Published:** 2022-12-08

**Authors:** Magdalena Skoworonska, Annika Blank, Irene Centeno, Caroline Hammer, Aurel Perren, Inti Zlobec, Tilman T Rau

**Affiliations:** ^1^ Institute of Pathology University of Bern Bern; ^2^ Institute of Pathology Triemli Hospital Zurich Switzerland; ^3^ Institute of Pathology University Hospital and Heinrich‐Heine‐University Düsseldorf Düsseldorf Germany

**Keywords:** biobanking, SPREC, cold ischemia time, warm ischemia time, fixation time, LEAN

## Abstract

The standardized preanalytical code (SPREC) aggregates warm ischemia (WIT), cold ischemia (CIT), and fixation times (FIT) in a precise format. Despite its growing importance underpinned by the European *in vitro* diagnostics regulation or broad preanalytical programs by the National Institutes of Health, little is known about its empirical occurrence in biobanked surgical specimen. In several steps, the Tissue Bank Bern achieved a fully informative SPREC code with insights from 10,555 CIT, 4,740 WIT, and 3,121 FIT values. During process optimization according to LEAN six sigma principles, we identified a dual role of the SPREC code as a sample characteristic and a traceable process parameter. With this preanalytical study, we outlined real‐life data in a variety of organs with specific differences in WIT, CIT, and FIT values. Furthermore, our FIT data indicate the potential to adapt the SPREC fixation toward concrete paraffin‐embedding time points and to extend its categories beyond 72 h due to weekend delays. Additionally, we identified dependencies of preanalytical variables from workload, daytime, and clinics that were actionable with LEAN process management. Thus, streamlined biobanking workflows during the day were significantly resilient to workload peaks, diminishing the turnaround times of native tissue processing (i.e. CIT) from 74.6 to 46.1 min under heavily stressed conditions. In conclusion, there are surgery‐specific preanalytics that are surgico‐pathologically limited even under process optimization, which might affect biomarker transfer from one entity to another. Beyond sample characteristics, SPREC coding is highly beneficial for tissue banks and Institutes of Pathology to track WIT, CIT, and FIT for process optimization and monitoring measurements.

## Introduction

Documentation of preanalytical variables for biological material has become increasingly important in recent years due to growing concerns about the reproducibility of research results in the domain of personalized medicine [1, 2]. This is also reflected in the *in vitro* diagnostics (IVD) regulation implemented by the European Union, which obliges companies to outline the applicable preanalytical framework for their diagnostic test. Pathology laboratories, which are at the source of the material, rarely track relevant time points for preanalytical data generation, or quantitatively outline the real‐life range of preanalytical variability in which robust biomarker development should function [3].

Preanalytical variables describe the processing of biomaterials before they enter the analytical phase in a research or clinical setting. Some of those variables have proven to have striking effects on readouts in the analysis of DNA [4, 5, 6, 7, 8, 9], RNA [8, 10, 11, 12, 13], proteins [14, 15, 16], posttranslational modifications [17, 18], and many more derivatives. The importance of preanalytics has been highlighted in huge collaborative projects like the Cancer Genome Project (TCGA), which necessitate samples with excellent RNA quality (using RNA integrity number [RIN] values as surrogate markers) on the one hand, and histopathological control requiring high tumor content on the other. Preanalytical programs launched by the National Institutes of Health (NIH) currently try to unravel the robustness and variability of biomarkers under different conditions [19]. These projects underpin the importance of quality issues for subsequent upscaled multiomics research [20] but conceptually miss one essential element: the standards of routine preanalytical data stemming from routine pathology laboratories.

The standard pre‐analytical code (SPREC) unifies the most critical variables potentially affecting each biospecimen in designated categories [21, 22]. For solid tissue, the code describes warm ischemia time (WIT), cold ischemia time (CIT), fixation time (FIT) and type, the duration of storage, and storage temperatures. International and national associations like the International Society for Biological and Environmental Repositories (ISBER), Biobanking and BioMolecular resources Research Infrastructure – European Research Infrastructure Consortium as well as, in our case, the Swiss Biobanking Platform (SBP) strongly support the adherence to SPREC. However, the arbitrarily defined ischemia and fixation times of the SPREC code are not corrected for their empirical occurrence [21]. This is due to a lack of knowledge about the SPREC variables in Institutes of Pathology and even biobanks. For instance, in 2017, none of the participating internationally advanced tissue banks in Switzerland were able to provide all the informative data required for a complete SPREC code, mostly failing in data about fixation times [23]. Tracking the starting time for the point of fixation in the surgical environment or during biopsy, and its end by tracking the paraffin‐embedding time point matters, but is rarely performed due to interdisciplinary obstacles or a lack of laboratory tracking systems.

Process optimization and monitoring that takes place in tissue banks regularly outperforms *ad hoc* self‐made collections in terms of quality management. Optimized processes help minimize some of the external influences on tissue quality by implementing procedures such as snap freezing within the surgical theater [24, 25], the use of particular fixatives [26], and the exclusion of inferior biospecimens. There are several methods and systems for laboratory process optimization. In our case, LEAN six sigma management was applied [27]. Few real‐life data have been published, but surgical and pathological grossing processes might form the limits of achievable SPREC values. Notably, research restricted to the highest quality samples could introduce biases toward preselected specimen types (e.g. short surgery times) and reflect less realistic sample processing from routine clinical settings.

The Tissue Bank Bern (TBB) successively integrated monitoring for clamping, resection, and fixation time points, thus giving access to the data of *n* > 12,500 process samples offering valuable information including routine formalin fixation times.

With this study, we aim to: (1) outline our real‐life preanalytical data set as a possible standard template for pathology institutes with frozen section units and (2) highlight how parameters such as workload, daytime, laboratory logistics, and organ‐specific differences can affect the dynamics of preanalytical variables.

## Materials and methods

### Organization

At the University Bern, biobanking was installed with the foundation of the TBB in 2003. The facility is hosted by the local Institute of Pathology and, together with the recently established liquid BioBank of the University Hospital Bern, forms the joined Biobank Bern. TBB is responsible for the collection, the processing, and documentation of newly delivered biospecimens for further use in research under the umbrella of a quality management system and the concept of Biobanking 3.0 [28]. The acquisition of samples is performed by trained residents, histopathology technicians, and employees of the TBB. Access to samples is governed through written applications, the contributing clinics, and the regulations by a local steering committee. Additional project‐related services are offered by the Translational Research Unit of the Institute of Pathology. TBB is attributed the OPTIMA label of the SBP, which relies on the ISO norm 20387 [29].

### Cohort description

**Figure 1:**
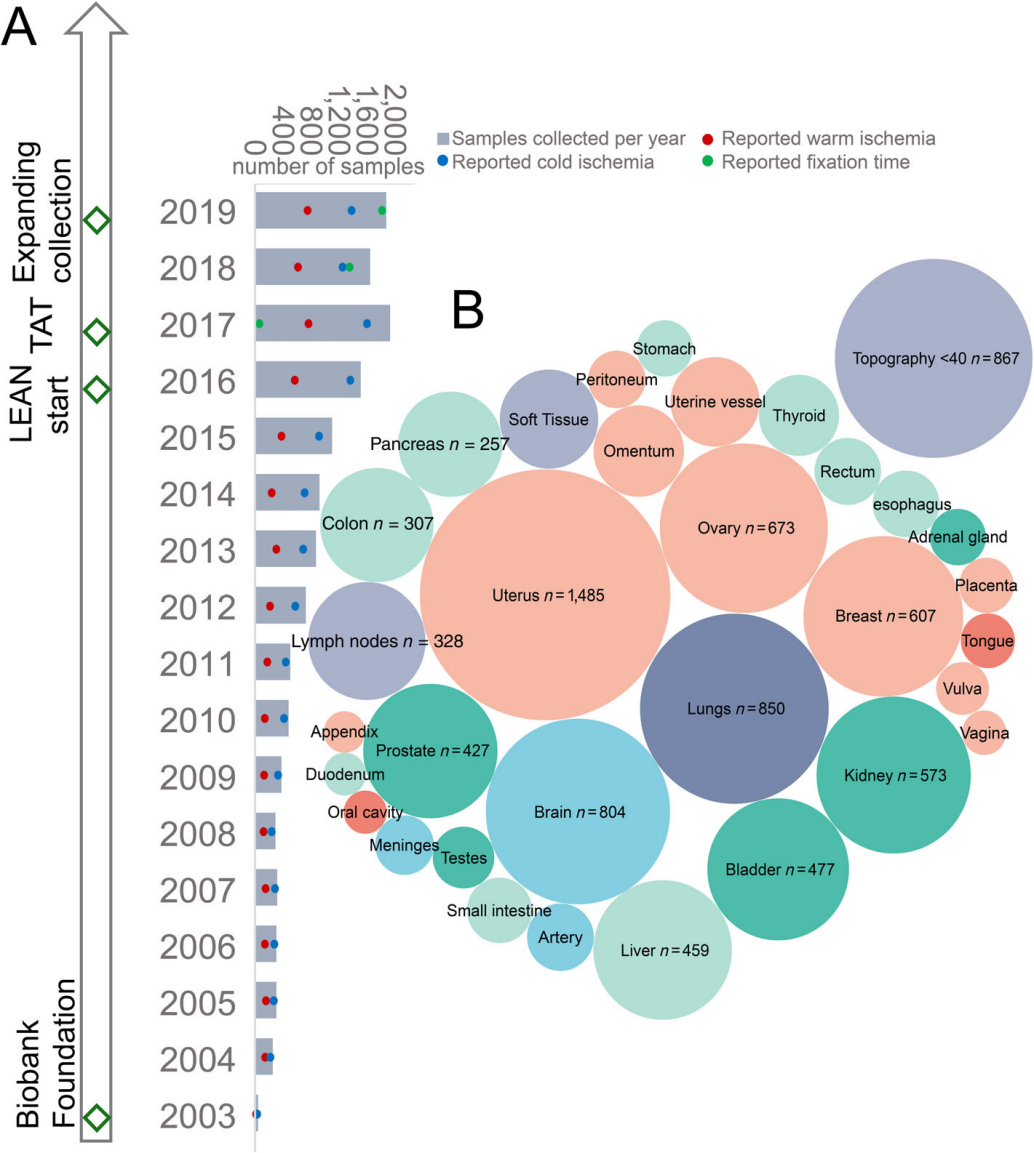
Growth and content of TBB. (A) Histogram of growth of collection in number of samples collected per year. Red dots indicate the coverage of available WIT, blue dots indicate CIT, and green dots indicate FIT. Major events affecting the biobank collection are highlighted with diamonds along the timescale; TAT, turnaround time. (B) Number of samples within the TBB sorted by topography, with bubble size corresponding to number of cases. The color grouping of bubbles shows origin from different clinics.

Data on biobanked specimens were gathered from 2003 to 2019. In total, 12,674 samples with complete data points were actionable. The number of departments contributing specimens for biobanking rose over time, providing a vast range of different topographies. In 2019, we collected ~1,800 different samples, which corresponds to ~5% of all histological diagnostics requests with mainly surgical resections as the source of specimens. The cohort can thus be regarded as representative of any Institute of Pathology providing frozen section or tissue biobanking facilities. Details of the collection are outlined in Figure 1 and Table 1.

**Table 1:** Sample characteristics

Feature	Freq *N* (%) or mean (range)
Total samples	12,674 (100%)
Department	
Gynecology	4,951 (39.1%)
Head and Neck	474 (3.7%)
Neurosurgery	1,178 (9.3%
Thoracic Surgery	1,346 (10.6%)
Urology	2,787 (22.0%)
Visceral Surgery	1,748 (13.8%)
Other	190 (1.5%)
Experience level	
Learning phase (up to 100 specimens provided)	790 (6.2%)
Experienced phase (>100 specimens provided)	11,884 (93.8%)
Processing day	
Monday	1,926 (15.2%)
Tuesday	3,434 (27.1%)
Wednesday	3,002 (23.7%)
Thursday	2,396 (18.9%)
Friday	1,910 (15.1%)
Saturday	4 (<1%)
Sunday	2 (<1%)
Processing hour	
8–9	227 (1.8%)
9–10	1,117 (8.8%)
10–11	1,567 (12.4%)
11–12	2,229 (17.6%)
12–13	2,190 (17.3%)
13–14	1,638 (12.9%)
14–15	1,305 (10.3%)
15–16	1,209 (9.5%)
16–17	748 (5.9%)
17–18	243 (1.9%)
Other	38 (<1%)
LEAN optimization	
Before	6,359 (50.2%)
After	6,315 (49.8%)
Workload	
Average per day	5.93 (1–19)
Average per month	101.65 (10–196)
Time stamps	
Surgical clamping	5,888 (46.5%)
Surgical resection	10,993 (86.7%)
Delivery from surgery	9,850 (77.7%)
Registration pathology	12,509 (98.7%)
Endpoint fresh processing	12,134 (95.7%)
Paraffin embedding	3,087 (24.4%)

### Time point definition of WIT, CIT, and FIT

The following time points are routinely collected at the TBB: clamping time (if applicable), resection time, delivery from surgical theater, reception time at pathology, end of fresh processing (freezing/start of fixation), and paraffin embedding (cessation of fixation). The first three values are dependent on interdisciplinary work with the contributing clinics, which originate these measurements. The three latter values were measured in the Pathology Department. WIT is defined as the difference between clamping and resection, and CIT extends from resection to the end of fresh tissue processing. FIT is defined as extending from the end of fresh processing until paraffin embedding for corresponding additional formalin‐fixed paraffin‐embedded (FFPE) blocks. The essential values of the SPREC code 3.0 are displayed in Table 2. Local time measurements were made to the minute using local radio‐controlled clocks at both sites (clinic and pathology) in the early years and then a shift toward computer‐based network time protocol time logs. Manual data entry was consistently based on paper notification and in 2015 shifted toward the electronic ordering system (Ixserv, ix.mid GmbH, Cologne, Germany).

**Table 2:** Essential parameters of the SPREC code 3.0 applicable to this study, incomplete excerpt from Betsou *et al* [21]

Feature	Category	Time
WIT	A	<2 min
	B	2–10 min
	C	10–20 min
	D	20–30 min
	E	30–60 min
	F	>60 min
	X	Unknown
CIT	A	RT <2 min
	B	RT 2–10 min
	C	RT 10–20 min
	D	RT 20–30 min
	E	RT 30–60 min
	F	RT 60 min to 3 h
	G	RT 3–6 h
	H	RT 6–12 h
	I	RT >12 h
	X	Unknown
FIT	A	<15 min
	B	15 min to 1 h
	C	1–4 h
	D	4–8 h
	E	8–24 h
	F	24–48 h
	G	48–72 h
	H	>72 h
	X	Unknown

*Note*: RT, room temperature.

### Data monitoring

Continual data monitoring took place on a weekly basis during the registration of a sample entry. Obvious mistakes in documentation (e.g. resulting in negative values in WIT, CIT, and FIT) were considered not applicable. Additionally, surgeries without blood clamping could not contribute to WIT data gathering. When single values were missing, WIT, CIT, and FIT could not be calculated. Detailed information about time stamp data collection can be taken from Table 1. In total, 10,555 CIT, 4,740 WIT, and 3,121 FIT values were accessible for this study.

### Process optimization with LEAN six sigma

LEAN optimization of the TBB took place in 2016. A thorough analysis of the internal processes took place according to the principles of six sigma (6S). We analyzed the complete biobanking process within the Institute of Pathology, University of Bern, from the resection in the surgical theater, reception of the specimen until long‐term storage with process description according to our local, national (SBP), and international (ISBER) quality standards. We identified influencing factors, workload peaks, and organizational aspects in order as the main bottlenecks for quick‐freezing processes. We optimized supporting platforms for measurements, with an additional fresh material working space, short distances to freezing, and regular training sessions for laboratory and medical staff, with staggered lunch breaks for the team, since many specimens were registered in the Pathology Department between 1100 and 1300 h. We standardized our processes toward achievable time frames with the goal of 20 min hands‐on time until final freezing in‐house to achieve a total CIT <1 h. We sustained this process by measuring turnaround times on a weekly basis and outlining the results for the team using a traffic light system for the average and longest CIT values. Outliers were treated as critical incidents and followed up in detail. Barcoding with tube systems (Fluid X, Azenta Life Sciences, Chelmsford, MA, USA) was introduced for safety, as well as working space equipment at S2‐level with masks and metal gloves as personal gear. Elements outside the Institute of Pathology such as intrasurgery optimization affecting WIT, or the logistics of transport affecting CIT, were not part of the LEAN process optimization.

### Data export and categories

Data entry takes place in the regular laboratory information system of the Institute of Pathology (PathoWin plus, Basys Data, Basel, Switzerland). This allows data export with differing parameters and includes information on SNOMED coded topographies of the biobanked organ, and the dates and contributing clinics.

### Statistics

Descriptive statistics regarding frequencies and percentages are provided as histograms and bubble charts. Correlations were calculated with Pearson's correlation coefficient. ANOVA and Student's *t*‐test were used to search for differences. Significance was outlined at *P* values of <0.05 and indicated with asterisks as follows: ***p < 0.001, **p < 0.001, and *p < 0.01.

## Results

### Types and amount of material collected in TBB

The material collection steadily increased from an average <300 samples per year in 5 years after inception to >1,500 samples in the last 5 years (Figure 1A). Associated preanalytical time points are available for ~37% of samples with regard to WIT – as this is not applicable for every surgical intervention – and ~83% for CIT, indicating a high documentation level of completed data points. In 2018, we introduced the tracking of FIT for corresponding FFPE blocks, thus providing FIT for ~24% of all specimens. Sampling was performed from surgically active clinics at the Bern University Hospital. The major contributions are gynecological, urological, and visceral surgery specimens (75%). In recent years, an increasing number of clinics followed the official tissue biobanking procedure on campus. The topography of samples and size of collection are highlighted in Figure 1B.

### Distribution patterns within the SPREC code variables

**Figure 2:**
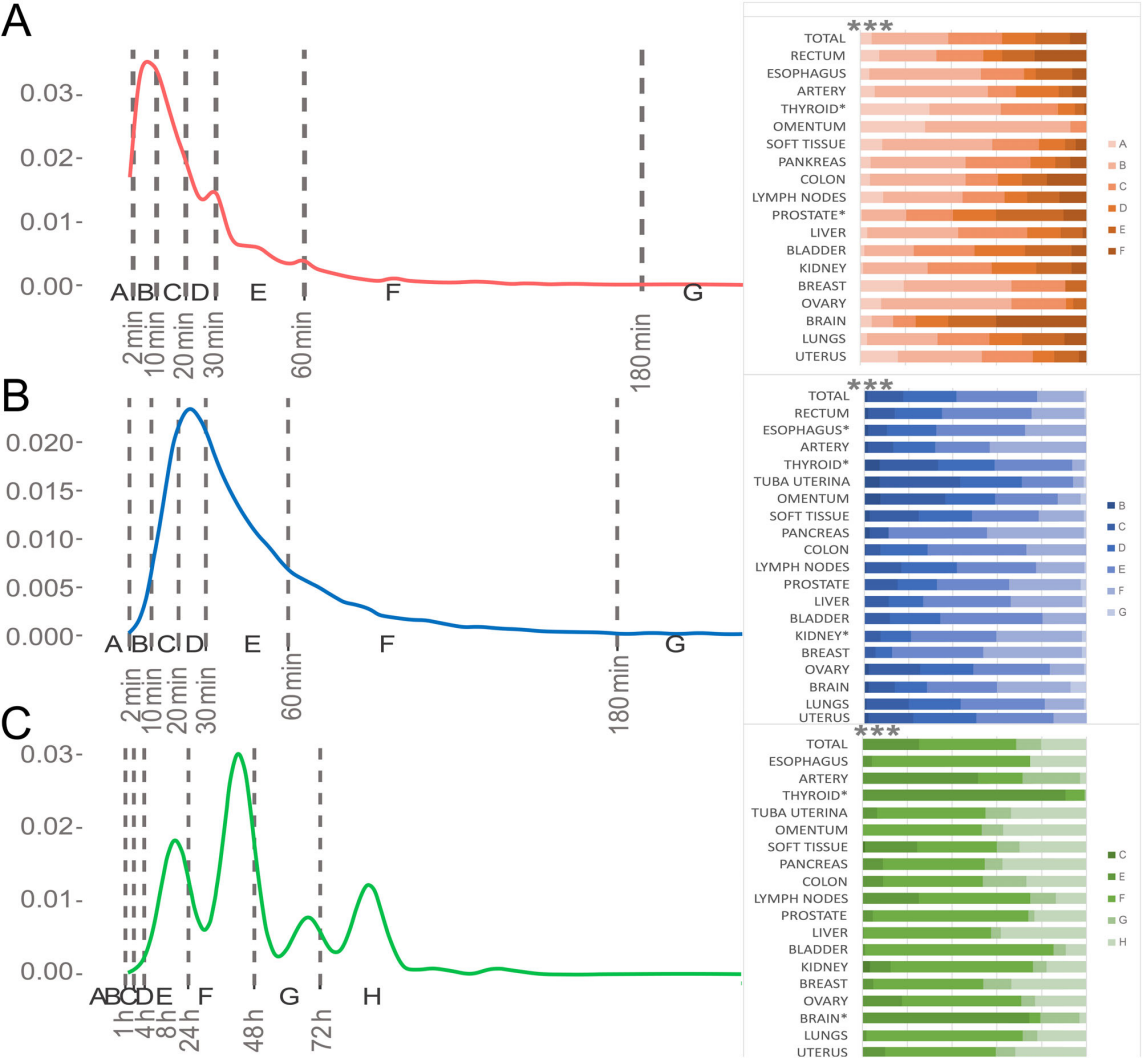
Distribution of ischemia and fixation times in total collection and among most common topographies. (A) WIT = red; (B) CIT = blue; and (C) FIT = green. SPREC in its defined categories is depicted along the timeline with gray horizontal lines marking the thresholds. The right part of the graph shows the distribution of SPREC code among different topographies. ANOVA across all specimens revealed significance for organ dependencies for WIT, CIT, and FIT, each *p* < 0.001, respectively (***).

The distribution of WIT, CIT, and FIT values from biobanked samples is represented in Figure 2. In brief, ascending letters indicate longer durations. For exact definitions, we refer to the SPREC code [21]. The WIT values in total are mainly distributed in SPREC categories B 34%, C 24%, D 15%, and E 15%; CIT of the samples fell mainly in the SPREC categories E 36%, D 24%, F 21%, and C 16%; while FIT showed the greatest spread of values across SPREC categories F 43.6%, E 25%, H 20%, and G 11%.

Under these real‐life conditions, SPREC showed a tendency toward standardized and optimized values for WIT and CIT for a surgery‐driven hospital‐integrated biobank, which can be appreciated with single peaks in the histogram. This does not apply to FIT values, however, which show tremendous undulations. Notably, the proposed thresholds within SPREC were chosen to represent working days (24, 48, and 72 h, E, F, and G, respectively) but do not take into consideration the tissue processing steps within the vacuum infiltration tissue processors, nor the stopping of fixation at paraffin embedding. This results in an appreciable shift of 4–6 h against the proposed SPREC timelines (Figure 2C).

### Distribution of core preanalytical values and organ dependency on ischemia times

Differences in surgical procedures explain WIT differences. Short access and removal times are observed for breast (mean WIT = 10.4 min) or thyroid glands (mean WIT = 12.1 min) in comparison to deep anatomic structures, for example, the prostate (mean WIT = 34.4 min) or rectum (mean WIT = 39.9 min).

Pathological grossing causes major differences in CIT depending on organ and tissue type. Short CIT occurs in superficial tumors such as oral carcinomas (mean CIT = 25.3 min) and the thyroid (mean CIT = 32.1 min). This is contrasted by prolonged processes due to inking, pinning, and initial grossing steps, for example, in esophagus (mean CIT = 48.9 min) and soft tissue tumors (mean CIT = 44.4 min).

FIT as a preanalytical variable is particularly dependent on tissue properties and clinical decision‐making. For instance, the thyroid gland has shorter fixation times and most of the samples fall into category E (91%). Process definition by the laboratory based on microtomic performance on specific tissue types is the main driver of different FIT schedules. In general, some tissues need to harden more (breast mean FIT = 63.2 h) or have properties as unique barriers (placenta mean FIT = 64.4 h), which significantly differ from wet (thyroid mean FIT = 21.9 h) or smaller samples (hypophysis mean FIT = 39.0 h). In summary, all three parameters of WIT, CIT, and FIT are highly dependent on topography (ANOVA *p* < 0.001 each, respectively).

### Weekday dependency of preanalytics

**Figure 3:**
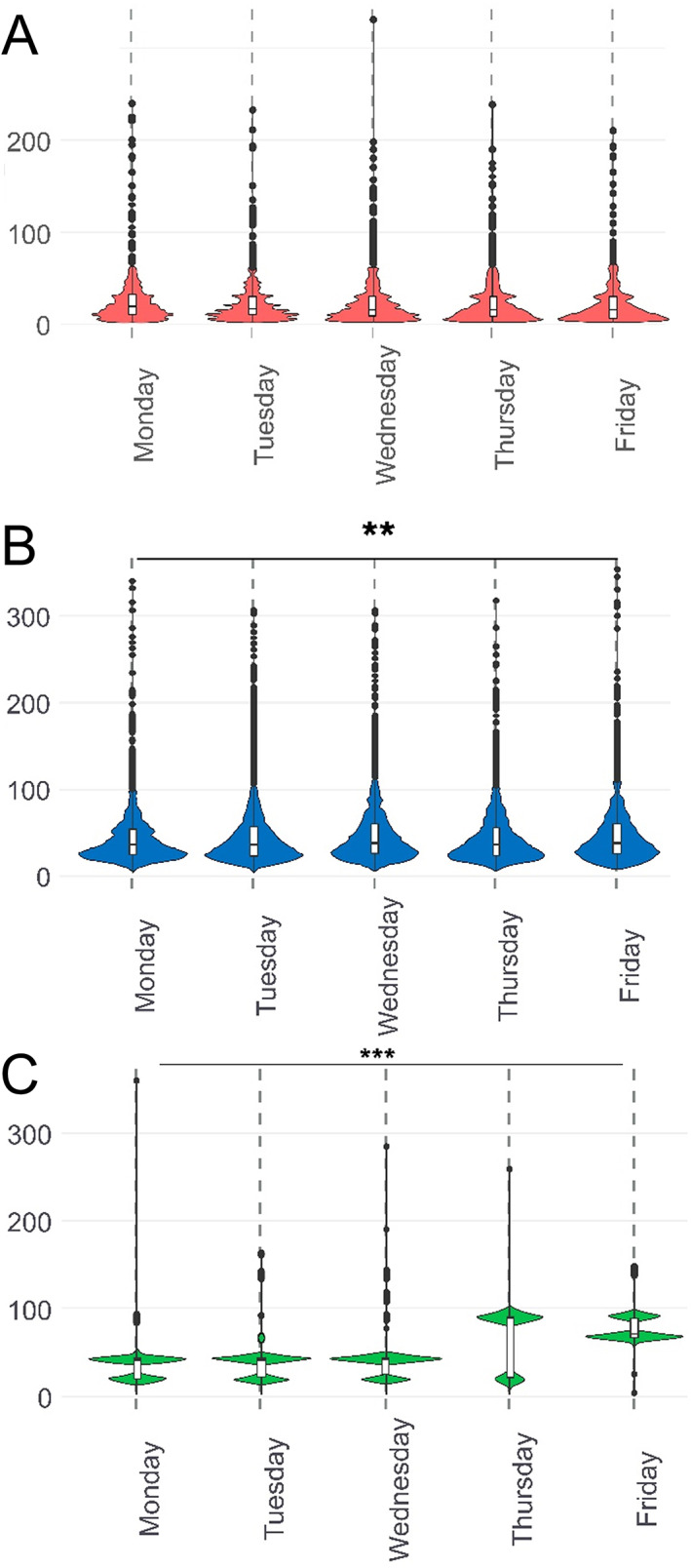
Dependency of ischemia and FIT by weekday. Violin plots for (A) WIT = red; (B) CIT = blue; and (C) FIT = green on different working days. ANOVA showed no significant differences for WIT but, to a very small but significant degree of 3 min, procrastination on Friday – **p = 0.001 for CIT. Notably, the biggest effects can be seen in FIT with regular ‘two‐peaked’ distributions and pronounced shifts close to weekends with significantly stretched FIT, ***p < 0.001. The shift on Thursdays is the biggest, as either next day processing or 72 h prolongation takes place.

Next, we tested for weekday dependencies. Most surgeries at our hospital are performed on Tuesdays and Wednesdays, leading to elevated burdens (Table 1), which translate into higher preanalytical values (see also workload dependency below). Differences in WIT and CIT resulted in very small but significant ranges from 23.6 to 26.3 min for WIT, 45.1 to 48.6 min for CIT across the week (ANOVA *p* < 0.001), which are only appreciable in the violin plots used to visualize the number of cases and corresponding times by weekday (Figure 3). Notably, fixation times behave completely differently now that same day processing in pathology is not taking place on a regular basis. This leads to a notably broad range of 35.8–76.4 h of fixation with two noticeable FIT peaks per day with a very strong effect of weekend processes. Interestingly, this also takes place on Thursdays, with the most stretched range of fixation hours. As expected, the differences in FIT are highly significant (ANOVA *p* < 0.001).

### Daytime dependency of preanalytics

In the hourly sample, registration time‐stamps, and workload peaks are obvious during lunchtime (defined from 1100 to 1400 h), in which 47.8% of samples are registered. The difference in CIT across the complete cohort analyzed shows an average 1.5 min delay during lunchtimes per specimen (*p* = 0.037), and although this is short, it should be seen in the context of a theoretical optimum of 20 min in‐house handling time. WIT and FIT were not significantly different over the course of the day.

### Preanalytical variables depending on clinic

**Figure 4:**
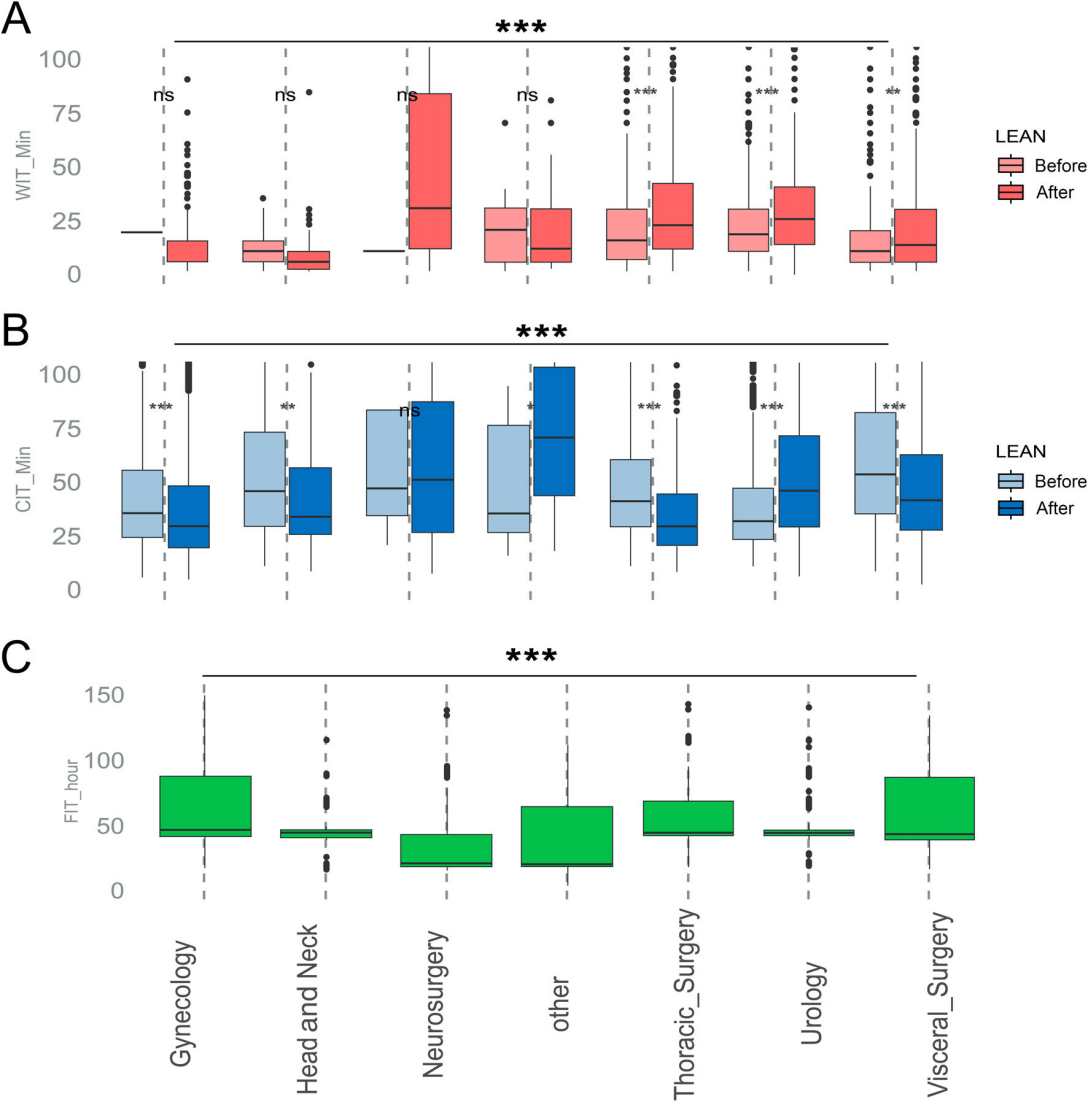
Dependency of ischemia and FIT from contributing clinics and LEAN effects. Boxplots highlight (A) WIT in minutes; (B) CIT in minutes; and (C) FIT in hours, averaged per main contributing clinics listed at the bottom of the graph. An additional separation in pre‐ and post‐LEAN measurements (before and after, respectively) can be appreciated for WIT and CIT data. As FIT data were introduced after LEAN optimization, only single boxplots are depicted per clinic. The asterisks indicate the *P* values from ANOVA tests for the complete set and Student's *t*‐test for side‐by‐side comparisons. Significances are shown as ***p < 0.001, **p = 0.001, and *p = 0.01, respectively.

Looking at differences of the contributing clinics and departments, the above‐mentioned organ‐specific effects affect the variables WIT, CIT, and FIT, but the process‐dependent logistics also come into play. In well‐established long‐standing clinical–pathology collaborations between clinics and the Pathology Department, the influence of LEAN management resulted in a significant decrease in cold ischemia. In our case, this applied to the Departments of Gynecology, Head and Neck, Visceral and Thorax Surgery (Figure 4).

Against this trend, we observed counter‐intuitive prolonged periods after LEAN implementation in other more recently contributing clinics such as neurosurgery as an outlier. In this particular case, clinical training in the assessment of time points (e.g. definition of resection status) or logistics had yet to be optimized. As a consequence, a training phase was defined for all new clinics, whereby the first *n* = 100 samples acted as a learning phase. These were removed from the following calculations.

LEAN process optimization was dedicated to the frozen section unit in pathology and did not cover intersectoral optimization. The focus should therefore be placed on CIT values (Figure 4). The CIT improvement was highly significant in the experienced collaboration phase with contributing clinics, from 47.9 to 45.2 min (Student's *t*‐test, *p* < 0.001).

### Reduced caseload dependency of preanalytics after LEAN optimization

Before LEAN introduction, the workload correlated positively with an extended CIT (*R* values 0.237, 0.358, 0.372 for daily, monthly, or yearly data, each *p* < 0.001). Short WIT inversely correlated with a higher workload due to the higher number of possible surgeries (*R* values −0.76; −0.103, −0.107 for daily, monthly, or yearly data, each *p* < 0.001). FIT had not been measured before LEAN process optimization.

Notably, this correlation for CIT disappeared after LEAN optimization (*R* values 0.000, 0.012, −0.06 for daily, monthly, or yearly data, each *p* > 0.05). The correlation between reduced WIT and more surgeries remained at lower levels on a daily basis after LEAN introduction (*R* value −0.065, *p* = 0.001). The additionally gathered fixation times could not be controlled against LEAN optimization but could be controlled against workload dependencies. Lower values for higher workloads (*R* values −0.233, −0.048, −0.097 for daily, monthly, or yearly data, each *p* < 0.001) indicated faster processing.

Moreover, we later used the mean case numbers per day (*n* = 6, range 1–19) to split the low and heavy workloads on a daily basis. Before LEAN optimization, the mean CIT differed between 43.9 min for daily low workload versus 74.6 min for days with a heavy workload: Student's *t*‐test, *p* < 0.001. After LEAN optimization, this difference with values of 43.7 min versus 46.1 min was negligible; Student's *t*‐test, *p* = 0.576. In summary, LEAN process management enabled the tissue biobank to be more resilient to higher volumes of incoming specimens and increased workloads (Figure 4).

## Discussion

### Dual role of ischemia times as a quality and process indicator

Achieving informative SPREC values for biobanks is a great challenge. SPREC outlines unknown parameters with an ‘X’. The tissue bank has progressed from the less informative SPREC ‘TIS‐SRG‐X‐X‐NBF‐X‐P’, which indicates a regular neutral buffered formalin‐fixed paraffin block from a surgical intervention, to a more informative state by completing all data for CIT, WIT, and FIT. The most prevalent SPREC code during our study was ‘TIS‐SRG‐B‐E‐NBF‐F‐P’, which adds 2–10 min WIT, 30–60 min CIT, and 24–48 h FIT to the former. Even if this value has been derived from a single center in this study, it could serve as a template for many other Institutes of Pathology that organize biobanking activities in association with frozen section units. It may also serve as an important target preanalytical range for legal administrative procedures such as the novel IVD regulations [3]. Biomarker development should show robustness for exactly these routine conditions.

As long as biobanks cannot provide a complete informative SPREC for every sample, preanalytical effects in biomarker research will never be known. Notably, systematic analysis with multiple sample taking per surgical specimen is currently taking place in a huge NIH series [19]. The study is conceptualized as an *ex vivo* experiment with the immediate aliquoting of material. Variations in WIT cannot be surgically planned. The envisioned time points of CIT include long values of up to 12 h, outside the good clinical practice of either quick delivery to a Pathology Department or fixation in the surgical theater. A regular setting for FIT is suggested as 10–12 h, which might apply to biopsy specimens, but might be two‐ to six‐fold longer for surgical specimens according to our data. Prolonged fixation times due to holidays or long transportation processes could also go beyond the highest value of 72 h. The proposed concept therefore neither corresponds to SPREC 3.0 nor to our real‐life data [19, 21].

A major finding of our study was that several factors affect WIT, CIT, and FIT data, such as workload, daytime, laboratory logistics, topography, and contributing clinics. A thorough awareness of differences for the diseases being investigated, and the site‐specific circumstances, is therefore crucial. Figure 2 could help interested researchers to identify prototypic SPREC codes for their target entity. As a consequence, individual SPREC labeling for every sample should take place and is an excellent quality parameter. This could be reported within amended REMARK tables for cohort descriptions [30].

In addition to the scientific value of SPREC, biobanks receive process‐related data with the continuous monitoring of WIT, CIT, and FIT. Outliers such as unrealistic WIT values, delayed time to freezing, and over‐fixation due to holidays and so on could immediately lead to behavioral and organizational change for biobanks.

### LEAN management for process improvements and standardization

Systematic measurements, which we undertook with LEAN management in the TBB tissue bank [27], could be considered for improvement. Our measurements focused on native tissue processing within the Institute of Pathology, at the University of Bern. As expected, it was mainly CIT values that could be changed. The sustainable streamlining of processes improved the CIT values substantially and particularly helped the biobank to cope with heavy workloads. Furthermore, process optimization could be planned beyond the Pathology Department and include the surgical theater and transportation systems. Technical innovations such as automatic formalin dispensing systems (e.g. UltraSAFE, Abacus DX, Auckland, New Zealand) will not only improve safety and quality in the surgical theater but also provide the further necessary time stamps to calculate realistic FIT values.

### Limitations of our study

Our study is limited to routine surgical resections at one hospital site and might depend on local organization and conditions of the healthcare system. Our biobank does not contain biopsy specimens, which are regularly processed very quickly and are exposed to the risk of under‐fixation or shift to predominantly alcoholic fixation [31]. Additionally, we are aware that particularly FIT might further vary if specimens from external medical departments are collected via long‐distance transportation or the postal system. Our study also did not systematically investigate biological parameters such as RIN values [32], phosphoprotein amounts [33], or hypoxia‐induced factors [16, 34] as known actionable biomarkers for preanalytic research. An outreach to other biobanks and Institutes of Pathology in a multicentric way is warranted to develop a picture close to routine healthcare processes.

### Real‐life data as a challenge for the SPREC code 3.0

According to our data, some SPREC code variables are very unlikely to occur in the routine setting of surgical specimens. Almost no *A* or *F* values occur for WIT, indicating a WIT below 2 min, or clamping times longer than 1 h. Direct resections without clamping, however, as well as biopsies, could be referred to group A with WIT below 2 min.

The same is true for CIT. Ultrasnap freezing such as category ‘A’ can be performed with additional measurements within the surgical theater, but it does not apply for routine pathology settings. We experienced the introduction of SPREC 3.0 as a challenge, because temperature was added as a separate parameter allowing for cooling to 4 °C instead of room temperature to CIT as time parameter. Interestingly, this feature is solved by adding a number ‘4’ after the main CIT category. Temperature monitoring would notably be interesting for FIT as well, which has yet to be integrated. From our point of view, temperature values should be kept separated from other parameters in order to be more meaningful. Our optimization steps resulted in no tissues beyond 6 h CIT. Categories H and I were therefore not applicable in our setting; however, late night surgeries with closed tissue banking facilities or accidental situations are possible. Again, these scenarios do not reflect typical surgical–pathological workflows, where immediate fixation is recommended over cooling only.

Our results indicate that FIT peaks might be better covered when adding 4 h on the top of SPREC thresholds longer than 1 day. A category of 4 days is warranted as particular Thursday samples might receive such long FIT values. In consequence, ‘E’ = 8–28 h; ‘F’ = 28–52 h; ‘G’ = 52–76 h; ‘H’ = 76–100 h; and a new ‘I’ >100 h would thus be recommended.

### Take home messages for biobanks and Institutes of Pathology

With this study, we show that fully informative SPREC implementation is possible for any Institute of Pathology. Whereas WIT and CIT could be organ‐specific optimized, the greatest variation will remain with FIT. This focus should be encountered by the novel legislation processes such as the European IVD regulations that demand specifications of preanalytical variables for biomarker testing [3]. As shown in this study, LEAN process management might be considered to reduce turnaround times by reducing waiting times, avoiding batch effects, and workload dependencies as sources of delay [27]. Establishing the documentation of relevant time points comes at low costs and will be more and more retrievable from laboratory information systems in an automated way. In summary, the SPREC code not only characterizes biospecimen but also provides biobanks and Institutes of Pathology with a highly valuable parameter for process optimization.

## Author contributions statement

MS performed data monitoring, statistical analysis, and graphical work. AB and CH conceptualized LEAN six sigma measurements and implementation into the laboratory workflow. ICR was responsible for primary data entry and data integrity. AP contributed to the study concept in his role as head of the Tissue Working Group of the Swiss Biobanking Platform and expert in LEAN management. IZ supported the study in her role as head of the Translational Research Unit and Tissue Bank Bern, and through statistical work. TTR conceptualized the investigation in his role as medical and ethical advisor of the Tissue Bank Bern. Manuscript preparation was mainly performed by MS and TTR. All authors approved the final version of the manuscript.

## Ethics statement

This analysis was approved by the local ethics committee (approval number 2019‐01940). High standards for sample care and processes are also ethically relevant. The Tissue Bank Bern achieved the OPTIMA label of the Swiss Biobanking Platform, which is closely equivalent to ISO norm 20387, until official authorities are ready for accreditation processes in Switzerland.

## Data Availability

The data of this study are available on request from the corresponding author. The data are not publicly available due to access regulations of the Tissue Bank Bern.

## References

[1] Begley CG , Ellis LM . Drug development: raise standards for preclinical cancer research. Nature 2012; 483: 531–533. 22460880 10.1038/483531a

[2] Prinz F , Schlange T , Asadullah K . Believe it or not: how much can we rely on published data on potential drug targets? Nat Rev Drug Discov 2011; 10: 712. 21892149 10.1038/nrd3439-c1

[3] Dagher G , Becker K‐F , Bonin S , *et al*. Pre‐analytical processes in medical diagnostics: new regulatory requirements and standards. N Biotechnol 2019; 52: 121–125. 31102798 10.1016/j.nbt.2019.05.002

[4] Atanesyan L , Steenkamer MJ , Horstman A , *et al*. Optimal fixation conditions and DNA extraction methods for MLPA analysis on FFPE tissue‐derived DNA. Am J Clin Pathol 2017; 147: 60–68. 28122725 10.1093/ajcp/aqw205 PMC5848216

[5] Janecka A , Adamczyk A , Gasinska A . Comparison of eight commercially available kits for DNA extraction from formalin‐fixed paraffin‐embedded tissues. Anal Biochem 2015; 476: 8–10. 25640584 10.1016/j.ab.2015.01.019

[6] Kofanova O , Bellora C , Frasquilho SG , *et al*. Standardization of the preanalytical phase of DNA extraction from fixed tissue for next‐generation sequencing analyses. N Biotechnol 2020; 54: 52–61. 31398512 10.1016/j.nbt.2019.07.005

[7] Nechifor‐Boilă AC , Loghin A , Vacariu V , *et al*. The storage period of the formalin‐fixed paraffin‐embedded tumor blocks does not influence the concentration and purity of the isolated DNA in a series of 83 renal and thyroid carcinomas. Rom J Morphol Embryol 2015; 56: 759–763. 26429169

[8] Patel PG , Selvarajah S , Guérard K‐P , *et al*. Reliability and performance of commercial RNA and DNA extraction kits for FFPE tissue cores. PLoS One 2017; 12: e0179732. 28640876 10.1371/journal.pone.0179732 PMC5480995

[9] Silk MT , Mikkilineni N , Silk TC , *et al*. Prospective evaluation of unprocessed core needle biopsy DNA and RNA yield from lung, liver, and kidney tumors: implications for cancer genomics. Anal Cell Pathol (Amst) 2018; 2018: 2898962. 30652067 10.1155/2018/2898962 PMC6311765

[10] Baena‐Del Valle JA , Zheng Q , Hicks JL , *et al*. Rapid loss of RNA detection by in situ hybridization in stored tissue blocks and preservation by cold storage of unstained slides. Am J Clin Pathol 2017; 148: 398–415. 29106457 10.1093/ajcp/aqx094 PMC5848261

[11] Choi Y , Kim A , Kim J , *et al*. Optimization of RNA extraction from formalin‐fixed paraffin‐embedded blocks for targeted next‐generation sequencing. J Breast Cancer 2017; 20: 393–399. 29285045 10.4048/jbc.2017.20.4.393 PMC5744000

[12] Poremba C , Uhlendorff J , Pfitzner BM , *et al*. Preanalytical variables and performance of diagnostic RNA‐based gene expression analysis in breast cancer. Virchows Arch 2014; 465: 409–417. 25218890 10.1007/s00428-014-1652-0 PMC4180906

[13] von Ahlfen S , Missel A , Bendrat K , *et al*. Determinants of RNA quality from FFPE samples. PLoS One 2007; 2: e1261. 18060057 10.1371/journal.pone.0001261 PMC2092395

[14] Gündisch S , Annaratone L , Beese C , *et al*. Critical roles of specimen type and temperature before and during fixation in the detection of phosphoproteins in breast cancer tissues. Lab Invest 2015; 95: 561–571. 25730369 10.1038/labinvest.2015.37 PMC4421866

[15] Luebker SA , Wojtkiewicz M , Koepsell SA . Two methods for proteomic analysis of formalin‐fixed, paraffin embedded tissue result in differential protein identification, data quality, and cost. Proteomics 2015; 15: 3744–3753. 26306679 10.1002/pmic.201500147

[16] Neumeister VM , Anagnostou V , Siddiqui S , *et al*. Quantitative assessment of effect of preanalytic cold ischemic time on protein expression in breast cancer tissues. J Natl Cancer Inst 2012; 104: 1815–1824. 23090068 10.1093/jnci/djs438 PMC3514166

[17] Theiss AP , Chafin D , Bauer DR , *et al*. Immunohistochemistry of colorectal cancer biomarker phosphorylation requires controlled tissue fixation. PLoS One 2014; 9: e113608. 25409462 10.1371/journal.pone.0113608 PMC4237459

[18] Wu Y , Gaskins J , Kong M , *et al*. Profiling the effects of short time‐course cold ischemia on tumor protein phosphorylation using a Bayesian approach. Biometrics 2018; 74: 331–341. 28742267 10.1111/biom.12742 PMC5992063

[19] Carithers LJ , Agarwal R , Guan P , *et al*. The Biospecimen Preanalytical Variables program: a multiassay comparison of effects of delay to fixation and fixation duration on nucleic acid quality. Arch Pathol Lab Med 2019; 143: 1106–1118. 30785788 10.5858/arpa.2018-0172-OA

[20] Check E . Cancer atlas maps out sample worries. Nature 2007; 447: 1036–1037. 17597721 10.1038/4471036a

[21] Betsou F , Bilbao R , Case J , *et al*. Standard PREanalytical code version 3.0. Biopreserv Biobank 2018; 16: 9–12. 29377712 10.1089/bio.2017.0109 PMC11708182

[22] Betsou F , Lehmann S , Ashton G , *et al*. Standard preanalytical coding for biospecimens: defining the sample PREanalytical code. Cancer Epidemiol Biomarkers Prev 2010; 19: 1004–1011. 20332280 10.1158/1055-9965.EPI-09-1268

[23] Swiss_Biobanking_Platform . WG Tissue – activity report & recommendations, 2017. [Accessed 9 November 2022]. Available from:. https://swissbiobanking.ch/website/wp‐content/uploads/2018/05/20180322_SBP_WGTissue_report.pdf

[24] Mock A , Rapp C , Warta R , *et al*. Impact of post‐surgical freezing delay on brain tumor metabolomics. Metabolomics 2019; 15: 78. 31087206 10.1007/s11306-019-1541-2

[25] Zheng H , Tao Y‐P , Chen F‐Q , *et al*. Temporary ischemia time before snap freezing is important for maintaining high‐integrity RNA in hepatocellular carcinoma tissues. Biopreserv Biobank 2019; 17: 425–432. 31025876 10.1089/bio.2019.0003

[26] Viertler C , Groelz D , Gündisch S , *et al*. A new technology for stabilization of biomolecules in tissues for combined histological and molecular analyses. J Mol Diagn 2012; 14: 458–466. 22749745 10.1016/j.jmoldx.2012.05.002

[27] Blank A , Dawson H , Hammer C , *et al*. Lean management in the pathology laboratory. Pathologe 2017; 38: 540–544. 29043447 10.1007/s00292-017-0388-4

[28] Simeon‐Dubach D , Watson P . Biobanking 3.0: evidence based and customer focused biobanking. Clin Biochem 2014; 47: 300–308. 24406300 10.1016/j.clinbiochem.2013.12.018

[29] De Blasio P , Biunno I . New challenges for biobanks: accreditation to the new ISO 20387:2018 standard specific for biobanks. BioTech (Basel) 2021; 10: 13. 35822767 10.3390/biotech10030013 PMC9245471

[30] Sauerbrei W , Taube SE , McShane LM , *et al*. Reporting recommendations for tumor marker prognostic studies (REMARK): an abridged explanation and elaboration. J Natl Cancer Inst 2018; 110: 803–811. 29873743 10.1093/jnci/djy088 PMC6093349

[31] Moatamed NA , Nanjangud G , Pucci R , *et al*. Effect of ischemic time, fixation time, and fixative type on HER2/neu immunohistochemical and fluorescence in situ hybridization results in breast cancer. Am J Clin Pathol 2011; 136: 754–761. 22031314 10.1309/AJCP99WZGBPKCXOQ

[32] Haynes HR , Killick‐Cole CL , Hares KM , *et al*. Evaluation of the quality of RNA extracted from archival FFPE glioblastoma and epilepsy surgical samples for gene expression assays. J Clin Pathol 2018; 71: 695–701. 29463577 10.1136/jclinpath-2017-204969

[33] Vassilakopoulou M , Parisi F , Siddiqui S , *et al*. Preanalytical variables and phosphoepitope expression in FFPE tissue: quantitative epitope assessment after variable cold ischemic time. Lab Invest 2015; 95: 334–341. 25418580 10.1038/labinvest.2014.139

[34] Havelund BM , Olsen DA , Andersen RF , *et al*. The influence of tissue ischemia on biomarker expression in colorectal cancer. Appl Immunohistochem Mol Morphol 2013; 21: 298–307. 23060299 10.1097/PAI.0b013e31826f4475

